# Stress and Problematic Smartphone Use Severity: Smartphone Use Frequency and Fear of Missing Out as Mediators

**DOI:** 10.3389/fpsyt.2021.659288

**Published:** 2021-06-01

**Authors:** Haibo Yang, Bingjie Liu, Jianwen Fang

**Affiliations:** ^1^Academy of Psychology and Behavior, Tianjin Normal University, Tianjin, China; ^2^School of Psychology and Cognitive Science, East China Normal University, Shanghai, China; ^3^Faculty of Psychology, Tianjin Normal University, Tianjin, China

**Keywords:** stress, problematic smartphone use, fear of missing out, depression, anxiety, Suf

## Abstract

Problematic smartphone use (PSU) has been linked with stress. Higher levels of stress likely increased problematic smartphone use. We investigated relations between stress, fear of missing out, and problematic smartphone use. The aim of the current study was to analyze the mediating role of fear of missing out (FOMO) and smartphone use frequency (SUF) between stress and PSU. We surveyed a broad sample of 2,276 Chinese undergraduate students in July 2019, using the FOMO Scale, Smartphone Addiction Scale-Short Version, Smartphone Use Frequency Scale, and Depression Anxiety Stress Scale-21. The results showed that stress was associated with PSU severity. Gender differences were found in PSU severity. Furthermore, FOMO was positively associated with SUF and PSU severity. Structural equation modeling demonstrated that FOMO acted as a mediator between stress and PSU severity. FOMO and SUF acted as a chain of mediators between stress and PSU severity. SUF did not account for relations between stress and PSU severity. The study indicates that FOMO may be an important variable accounting for why some people with increased stress levels may overuse their smartphones.

## Introduction

Smartphone has become an integral part of people's lives, and people engage in many different types of activities (e.g., gaming, mobile payments, and social networking) on their smartphones (1, 2). A series of interactive behaviors with one's smartphone has become part of the routine for most individuals, which is ubiquitous, especially for young people (3, 4). The use of smartphones has greatly facilitated our lives. However, the emergence of smartphone use can create some negative effects, such as problematic smartphone use (PSU). The prevalence of PSU among Chinese undergraduates was estimated to be 21.3% (5). The current study focuses on the impact of stress, common among undergraduates, on PSU severity, and the mediating roles of fear of missing out (FOMO) and smartphone use frequency (SUF).

### Background on Problematic Smartphone Use and Stress, Fear of Missing Out

Problematic smartphone use (PSU) refers to the excessive use of smartphones with associated dysfunction, withdrawal difficulties, and other phenomena similar to substance addiction (6, 7). Studies have shown that prolonged use of mobile phones can cause cervical back and neck pain (8), increased risk of car accidents (9), and delay and impairment in academic and work performance (10). In addition, PSU is also highly correlated with anxiety, depression, and other mental health symptoms (7, 11). In previous studies, this construct has been similarly labeled as “smartphone addiction,” and “excessive smartphone use” (12). Smartphone addiction should describe a pathological symptom, and we are only measuring a relative intensity, not a criterion to classify addiction; it is not applicable. Excessive smartphone use should only describe smartphone use, and excessive smartphone use is not necessarily a problem. Therefore, we think PSU is the most suitable in this study.

Stress is an agitated state arising from a lack of means to attain the many social environmental demands that place a burden on an individual's typical ability to adapt (13). Stress can change people's physical and mental state, and people are more prone to addictive behavior in stressful situations (14–18). With the popularity of smartphones, more people use electronic devices to engage in social networking sites and watch videos to relieve stress when they are under pressure, which often brings negative consequences (19–21). Jie et al. (22) reported that stress from interpersonal relationships, school-related problems, and anxiety symptoms were significantly associated with excessive Internet use. Stress and PSU severity are also closely related [reviewed in Vahedi and Saiphoo (23)] with specific studies demonstrating such a relationship (24–26). In fact, social stress (26) and emotional stress (24) positively influence PSU severity. Furthermore, Cho et al. (25) demonstrated that stress had a significant influence on PSU severity in adults. As stress increases, self-control decreases, which leads to increased PSU severity (25). Therefore, we pose the following first hypothesis.

H1: Stress should be positively associated with PSU symptoms.

Studies have shown that the prevalence of PSU is about 30% among men and 29% among women (25). Factors linked to PSU among male college students include gaming app use, anxiety, and poor sleep quality (27). PSU among female college students has been linked to the use of multimedia apps, social networking services, depression, anxiety, and poor sleep quality (28). Both stress and gender can play a role in PSU (26, 29, 30). Social stress positively influences problem smartphone use, and women experience more social stress and use smartphones more for social purposes than men, and thus, women are more likely to develop habitual or addictive smartphone behaviors (26).

H2: Women should evidence greater levels of PSU.

FOMO is defined as a pervasive worry that others might be having rewarding experiences from which one is not part of and the desire to stay connected with what others are doing continually (31). FOMO involves anxiety about missing out on learning that others have experienced valuable experiences and a desire to maintain ongoing connections with others (31). SUF means the frequency of smartphone use. Many studies have found that FOMO has a close relationship with SUF and PSU severity. People who score higher in FOMO are more likely to overuse their smartphones to satisfy the desire to stay connected (32). FOMO and PSU are positively correlated, and higher FOMO can be a driver of PSU (33). A study by Elhai et al. (34) found that FOMO is closely related to negative emotions, social use of smartphones, and PSU severity. Also, FOMO and greater SUF were related to PSU severity. FOMO was associated with increased SUF (a small effect) and PSU (a large effect) (7). FOMO was also found most closely related to PSU severity and social stress (8).

H3: FOMO is positively associated with SUF (H3a) and PSU severity (H3b).

FOMO and SUF play an important mediating role between stress and PSU. Many studies have shown that anxiety and depression have important effects on problematic Internet and smartphone use (35–37). One study found that social anxiety and loneliness were significantly correlated with excessive use of online games, but when stress levels were controlled, the significant relationship disappeared (36). This finding shows that stress plays a significant role in overuse of the Internet. Studies have shown that stress is directly associated with PSU severity (24–26). At the same time, other psychological factors may play an important role between stress and PSU. For example, a study found that all predictors of Internet overuse lost statistical significance, including the effect of stress on online game overuse, after controlling for avoidance motivation and achievement motivation (36). Therefore, the SUF may play a mediating role between stress and PSU severity.

In addition, many empirical studies have examined the mediating role of FoMO in the relationship between psychological variables and PSU. For example, many studies have found that FOMO mediates relations between negative emotions (such as anxiety and depression) and PSU severity (35, 37). It has also been found that FOMO plays a mediating role in maximization and PSU (33). Therefore, we can assume that FOMO plays a mediating role between stress and PSU severity. Meanwhile, many studies have shown that elevated FOMO and elevated SUF are positively correlated, and SUF is a significant predictor of PSU severity (35). Therefore, we infer that there is another pathway that FOMO and SUF play a chain mediating role between stress and PSU severity. It is high FOMO and high SUF at high stress levels that lead to PSU.

H4: FOMO acts as a mediator between stress and PSU severity.
H5: SUF acts as a mediator between stress and PSU severity.
H6: FOMO and SUF act as a chain mediator between stress and PSU severity.

### Theory

The uses and gratifications theory (UGT) (38) was an early theory based on mass communication research to explain why people use media. According to this theory, people use particular types of media to satisfy specific needs they have. For example, individuals who feel lonely can use social apps to meet their social needs by interacting with friends or strangers. For this study, this theory may also explain the relationship between stress and PSU severity. For example, increased stress may lead individuals to use their smartphone for recreation and, thus, make themselves feel temporarily happy and relaxed. Meanwhile, an important characteristic of FOMO is the need to stay in constant contact with what others are doing (31). Przybylski et al. (31) argue that FOMO stems from a lack of need satisfaction, such as the need for social connection, and the use of smartphones allows people to get frequent social networking sites to get the status of life of people they follow, updates, and social hotspots, and to get satisfaction by doing so. Previous studies have also shown that individuals with high levels of FOMO have higher levels of SUF and PSU severity (27, 39, 40). Therefore, the UGT theory may be able to explain this phenomenon.

The compensatory Internet use theory (CIUT) (20) is a theory proposed for excessive Internet use. The CIUT suggests that when individuals face adversity (such as stress and negative emotions), they often use the Internet to relieve negative emotions such as stress, although this may adversely lead to Internet overuse. Nowadays, smartphones are so common and available that when people are unhappy and under pressure, they often unconsciously unlock their phones, watch a video, surf social networking sites, or play games (28), which also lead to increased PSU severity. CIUT theory is supported by empirical studies in PSU research (30).

The Interaction of Person-Affect-Cognition-Execution (I-PACE) model (41) is a comprehensive theory explaining problematic Internet use. This theory describes the process of developing excessive Internet use, involving a cycle, from core traits (such as genetic, biological, social cognition, personality, and specific motivation), to the subjective perception of emotional and cognitive reactions, the decision to use the Internet, and then obtain satisfaction, in turn, affecting the core traits. Each step of the process is closely related to whether it ultimately leads to problematic Internet use. The updated I-PACE model (42) has become more sophisticated, and suggests that the development of addictive behaviors is the result of interactions between inducing variables, emotional and cognitive responses to specific stimuli, and executive functions such as inhibitory control and decision making. The stages of Internet overuse were divided into early and late stages, and corresponding brain mechanisms were summarized. In I-PACE, FOMO is a prominent response variable to personal factors. It has been suggested that FOMO is well-suited as a response variable in I-PACE, representing a cognitive or affective bias mediating variable between personal factors and excessive Internet use (37, 43). Recent studies have found that FOMO mediates the relationship between negative emotions such as anxiety and depression and PSU severity (34, 43).

## Methods

### Procedure

We conducted an online survey at Tianjin Normal University in the fall of 2018 and spring of 2019. Institutional Review Board approval was first granted by the university. The university's Psychology Department recruited student participants through local online information on college bulletin boards and social networking accounts. These participants were directed to an informed consent statement and (for those who agreed) an online survey on wjx.cn, a Chinese online survey platform. All tests were conducted in Mandarin Chinese. There were 2,278 people who enrolled, but 15 participants who reported being younger than 15, or older than 27, were excluded. We also removed participants whose response time was substantially short or long. The remaining sampled included 2,263 participants, with an effective rate of 99.34% of those enrolling.

### Participants

Among the 2,263 participants, the average age was 19.35 years (SD = 1.36). A majority were women (*n* = 1,666; 73.6%), with 597 (26.4%) men. Most were of Chinese Han ethnicity (*n* = 2,075; 91.7%). A majority were freshman (*n* = 1,302, 57.5%) or sophomores (*n* = 669, 29.6%). Most were majoring in social/natural sciences (*n* = 1,668, 73.7%), language/humanities (*n* = 258, 11.4%), or engineering (*n* = 198, 8.7%). A majority reported being single/not in a romantic relationship (*n* = 1,699, 75.1%), with 541 (23.9%) participants in a relationship but not married.

### Instruments

#### Demographics

We queried gender, age, grade, race/ethnicity, relationship status, major, and years of smartphone use. Subsequently, the following psychological scales were administered.

#### Smartphone Use Frequency Scale

The SUF (44) was developed as an 11-item measure querying frequency of using specific smartphone features, with response options from 1 = Never to 6 = Very often. The features queried included “video and voice calls (making and receiving),” “text/instant messaging (sending and receiving),” “email (sending and receiving),” “social networking sites,” “Internet/websites,” “games,” “music/podcasts/radio,” “taking pictures or videos,” “watching videos/TV/movies,” “reading books/magazines,” and “maps/navigation.” We used the Chinese scale version, translated and validated previously, adding a 12th item tailored to this population: “educational learning.” Internal reliability for the Chinese scale is adequate (35). Cronbach's alpha in our sample was 0.819.

#### Depression Anxiety Stress Scale-21

Stress was measured by the Chinese version (17) of the 21-item DASS-21 (45). Each subscale is measured by seven items rated over the past week, with options from 0 = Did not apply to me to 3 = Applied to me very much or most of the time. We used the entire DASS-21 questionnaire for measurement, but the scores for stress in the model were scores for the dimension stress only. Internal consistency for the stress scale in this sample was 0.889.

#### Fear of Missing Out Scale

FOMO was measured by the Fear of Missing Out scale (FOMO) (31), which consists of 10 items (e.g., “I get anxious when I don't know what my friends are up to.”). Each item was rated from “1 = Not at all true of me” to “5 = Extremely true of me,” with higher total scores indicating higher levels of FOMO. We used the Chinese version, translated and validated previously (46). In the present study, Cronbach's α for the scale was 0.892.

#### Smartphone Addiction Scale—Short Version

We used the SAS-SV (47) to measure the severity of PSU by self-report, tapping health and social impairment, withdrawal, and tolerance components. The SAS- SV is the short version of the original SAS (47). The SAS-SV contains 10 items, with responses ranging from 1 = Strongly disagree to 6 = Strongly agree. Studies confirmed the reliability of the scale (48). A higher score means a higher degree of PSU. We used the Chinese version, which was previously translated and supported (28, 35). Cronbach's alpha in this study was 0.924.

### Data Analysis

SPSS 23.0 software was used for data processing, correlation, and descriptive analysis. We had no missing item-level data, as the web survey prompted participants to input responses for skipped items. We summed each scale's items for total scores. Scale scores were normally distributed, with the largest value for skewness being 1.12 (Stress) and for kurtosis being 1.99 (SUF).

**Figure 1:**
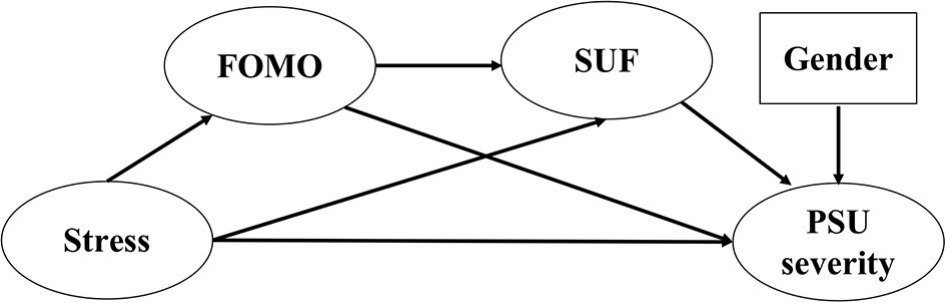
Hypothetical model diagram.

We used Mplus version 7 (Muthén & Muthén, 1998–2019) for confirmatory factor analytic (CFA) and structural equation modeling (SEM) analyses. We performed CFA for each scale in Figure 1, using item-level data, in order to test the scale's factor structure. We treated each scale's items as ordinal, using polychoric covariance matrices, weighted least squares estimation with a mean- and variance-adjusted chi-square (WLSMV), and probit-based factor loadings (49). Residual covariances were fixed to zero; all factor loadings were freely estimated, with factor variances fixed to a value of 1. We report fit indices including the comparative fit index (CFI) and Tucker–Lewis index (adequate fit between 0.90 and 0.94; excellent fit >0.94), standardized root mean squared residual (SRMR; adequate fit <0.08, excellent fit <0.05), and root mean square error of approximation (RMSEA, adequate fit from 0.07 to 0.08; excellent fit <0.07) (50).

We tested the hypothetical model in Figure 1. We discuss our use of latent variables below. The path from stress to PSU severity tests H1. The path from FOMO to PSU tests H3. The path from FOMO to SUF tests H4.

We tested mediation, computing the cross-product of direct path coefficients. We estimated standard errors for indirect (mediation) path coefficients using the delta method, with 1,000 bootstrapped, non-parametric samplings (51). We tested SUF as a mediator between FOMO and PSU severity (H5). We tested FOMO as a mediator between stress with PSU (H6). Finally, we tested FOMO as a mediator between stress with SUF (H6).

## Results

### Descriptive Statistics and Correlations

The descriptive and correlational results are shown in Table 1. The results showed that correlations between stress, FOMO, SUF, and PSU severity between the two reached a significant level (*p* < 0.001). The correlation between gender and SUF, and between gender and PSU severity reached a significant level (*p* < 0.01).

**Table 1:** Mean, standard deviation, and correlation analysis of each variable.

	**M**	**SD**	**1**	**2**	**3**	**4**	**5**
Stress	5.81	4.63	–				
FOMO	24.64	7.65	0.55^***^	–			
SUF	50.07	9.11	0.15^***^	0.22^***^	–		
PSU severity	36.25	10.94	0.49^***^	0.37^***^	0.35^***^	–	
Gender	1.74	0.44	−0.03	0.00	0.10^**^	0.15^**^	–

*FOMO, Fear of Missing Out Scale; PSU, problematic smartphone use; SUF, smartphone use frequency*.

** *p < 0.01*;

*** *p < 0.001*.

### Analysis of the Difference Test on Gender

Gender difference tests were done for PSU severity, SUF, stress, and FOMO. It was found that there were gender differences in PSU severity and SUF, and females scored significantly higher than males in PSU severity and SUF (*p* < 0.001). The gender differences in the scores of stress and FOMO were not significant (see Table 2).

**Table 2:** *T*-test for each variable on gender.

	**Male**	**Female**	***t***	***p***
PSU severity	33.52	37.23	51.83	0.000
SUF	48.49	50.64	24.57	0.000
Stress	6.05	5.72	2.20	0.138
FOMO	24.61	24.64	0.01	0.920

### SEM Results

Since gender has a significant influence on PSU, we controlled PSU for gender (8). There are three mediating pathways—one sequence mediating and two parallel mediating paths. The first parallel mediating path is stress → FOMO → PSU; The second parallel mediating path is stress → SUF → PSU. The sequence mediating path is stress → FOMO → SUF → PSU.

Structural equation modeling showed that the hypothesized model yielded a good fit, χ^2^(113) = 1070.079, χ^2^/df = 9.47, RMSEA = 0.061, SRMR = 0.040, CFI = 0.959, and TLI = 0.951. In general, if χ^2^/df > 3.84, RMSEA <0.08, SRMR <0.05, and CFI/TLI > 0.90, the structural equation model may be supported. In this study, the model fits the data well, which confirmed the multiple pathways model. We next found from the model path diagram (as shown in Table 3) that, except for the path “stress → SUF,” all the other paths reached significance (*p* < 0.05).

**Table 3:** Mediating effect tests.

**Path**	**β**	**SE**	***z***	**95% CI of β**	***p***
Gender → PSU (direct effect)	0.13	0.020	6.774	0.231:0.421	0.000
Stress → PSU (direct effect)	0.45	0.026	17.186	0.790:0.988	0.000
Stress → FOMO → PSU	0.04	0.016	2.296	0.005:0.068	0.022
Stress → SUF → PSU	0.01	0.009	0.880	−0.008:0.025	0.379
Stress → FOMO → SUF → PSU	0.04	0.007	5.554	0.026:0.053	0.000
Total mediating effect	0.08	0.017	4.854	0.051:0.117	0.000
Total effect	0.53				

**Figure 2:**
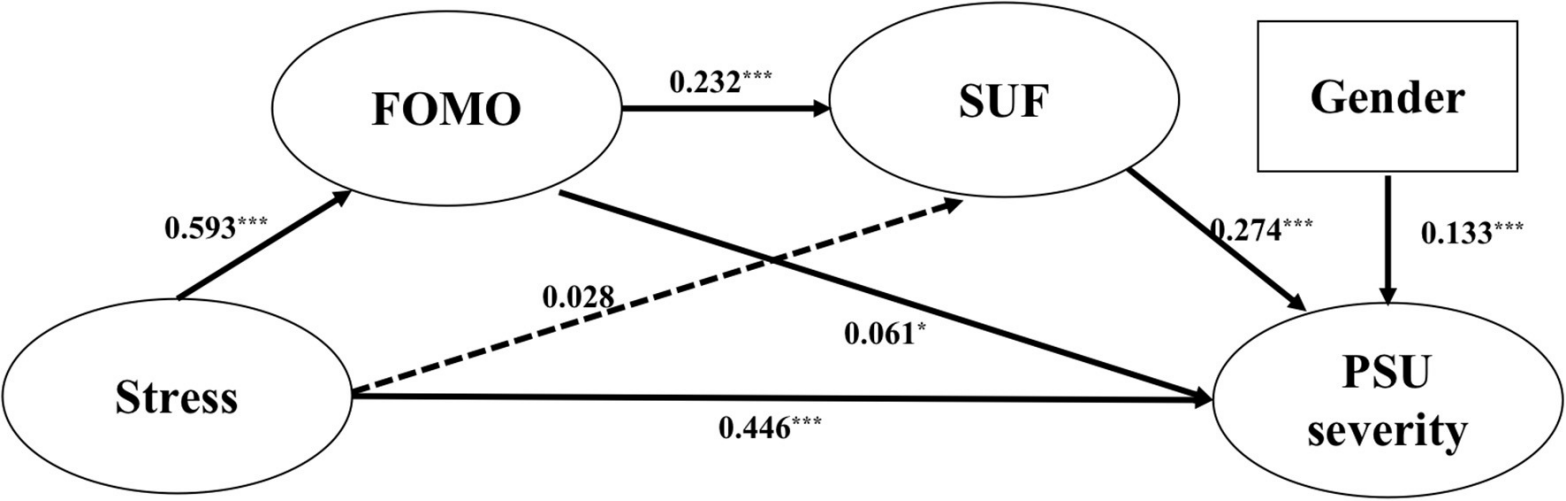
Results of the structural equation model. *p < 0.05; ***p < 0.001.

The results of bootstrapping for deviation correction (as shown in Table 3 and Figure 2) showed that both the direct effect and total mediating effect reached a significant level *p* < 0.001). In addition, the 95% confidence interval of bootstrap did not include 0, indicating that both the mediating effect and direct effect were supported.

The mediating effect of SUF between stress and PSU severity was 0.01, and the confidence interval contains 0, so the mediating effect was not significant. The mediating effect of FOMO between stress and PSU was 0.04, and the confidence interval does not contain 0, so the mediating effect was significant. The effect size for the mediation sequence between stress and PSU by FOMO and SUF was 0.08, and the confidence interval does not contain 0, indicating that this mediation sequence was significant.

## Discussion

The results of the study provide support that stress is associated with PSU severity in Chinese undergraduate students, supporting H1. Gender correlated with PSU severity, supporting H2. FOMO was positively associated with SUF and PSU severity, supporting H3. FOMO acted as a mediator between stress and PSU severity, supporting H4. FOMO and SUF acted as a chain of mediators between stress and PSU severity, supporting H6. Finally, SUF did not mediate relations between stress and PSU severity, thus rejecting H5.

Stress can predict PSU severity, supporting H1. This finding is in line with the CIUT (20) and UGT (38) that people will use smartphones more to meet their needs in the face of life adversity (e.g., high pressure), which may produce poor consequences. This result is also consistent with previous studies, which showed that people with higher stress use the Internet as a coping mechanism to relieve stress, thus, more likely to generate problematic Internet use (24–26, 52). The results of a systematic review suggest that stress is positively correlated with PSU severity use, with a small-to-moderate effect ranging from *r* = 0.20 to *r* = 0.30 (11). However, in our study, the correlation between stress and PSU was as high as 0.49, which may be related to the cultural phenomenon that Chinese college students have higher academic pressure from their parents and family (53) compared with students from other countries. One study has shown that the prevalence of PSU among Chinese college students is as high as 21.3%, and high stress is one of the risk factors for PSU among Chinese college students (5). Therefore, in Chinese college students, the relationship between stress and PSU may be stronger than in other cultural groups.

Gender was associated with PSU severity, supporting H2. Previous studies have shown that men and women differ in the degree of PSU and upper emphasis on smartphone use (54). Women are more likely to use social activities, while men are more likely to use procedural apps (such as games) (26). Overall, women spend more time on their smartphones. However, one study found that men had greater PSU severity (55). Nevertheless, in our study, we found that women had a higher degree of PSU severity. In order to more clearly verify the role of FOMO in the stress–PSU relationship, we controlled for the influence of gender on the degree of PSU severity.

The results showed that FOMO and SUF, FOMO, and PSU were significantly and positively correlated, supporting H3a and H3b. FOMO is a newly emerging and important psychological construct closely correlated with SUF and PSU severity (8, 27, 31, 39). Studies have shown that FOMO is highly correlated with negative emotions (such as anxiety and depression) (30). In this study, FOMO showed a moderate correlation with stress, *r* = 0.55. According to UGT (38), smartphones are used to satisfy their specific needs, so when individuals experience stress, they need to use certain features of smartphones to satisfy their needs to relieve stress and pursue relaxation and happiness. Also, high FOMO motivates individuals to use smartphones to satisfy the need to worry about missing cell phone messages, important news, etc. According to CIUT (20), when individuals face adversity (such as stress and negative emotions), they tend to use the Internet to relieve negative emotions such as stress. FOMO correlates with SUF and PSU severity, possibly because FOMO has components involving negative emotions. Previous studies have shown that FOMO was a predictor of SUF and PSU severity (35, 39, 40), which is consistent with our findings.

FOMO played a mediating role between stress and PSU severity, supporting H4. The result is consistent with the I-PACE model, proposing cognitive or affective bias variables such as FOMO (37, 41, 43) as mediating between subjectively perceived situations (stress) and problematic Internet use (37, 41, 42). When studying the relationship between levels of stress and PSU severity previously, studies often treated stress similarly to depression and anxiety (35), but there are differences. In the I-PACE model, stress is in a different position from anxiety and depression. Stress belongs to subjectively perceived situations, while anxiety and depression belong to the pathological components of an individual's core traits. Therefore, treating stress differently from anxiety and depression can help us better understand the important role of stress in PSU. Studies have shown that FOMO mediated relations between psychopathology symptoms (such as depression/anxiety) and PSU severity (35, 56). However, a few studies have been conducted on the relationship between FOMO and both subjectively perceived situations and PSU. Our research explored the relationship between the subjectively perceived situation and PSU severity and found that FOMO played a mediating role between stress and PSU severity, which fills the gap in this field and further verifies and expands the I-PACE model.

FOMO and SUF acted as a chain-mediating sequence between stress and PSU severity, supporting H6. The results are consistent with the I-PACE model, proposing cognitive bias variables such as FOMO (37) and also examining SUF mediating between subjectively perceived situations (stress) and problematic Internet use (41, 42). The reason is that the influence of FOMO on stress may be manifested by the increasing and habitual frequency of using mobile phones to form PSU (57).

However, surprisingly, the mediating path of stress to PSU severity via SUF was not significant, which is inconsistent with our hypothesis (H5). The results are also inconsistent with UGT's (38) conceptualization that individuals will seek satisfaction from media in the face of negative events. The findings suggest that stress does not directly lead to PSU severity by increasing SUF. Because unlike anxiety and depression, which are mental disorders, milder amounts of stress can be positive in some situations. For example, when individuals face deadlines, the sense of pressure may improve their work efficiency, so that they can complete the task in a short time with high quality. However, FOMO may play an important role in the relationship between stress and PSU. This conceptualization supports CIUT (20). In the empirical study that proposed this theory, stress did not directly lead to excessive Internet use, but required the mediating effect of motivation (36). The increase in pressure does not directly lead to increased SUF, which is also consistent with the I-Pace model. Since stress is a subjective feeling generated by an individual toward the external environment, cognitive and emotional responses are also needed between the stress and the decision to use a smartphone (41).

The present study had several limitations. First, although using a relatively large sample of undergraduates, all participants were from a single university in China, limiting generalizability regarding other countries. Second, the design was cross-sectional, and we cannot conclude that variables such as stress “predicted” or “caused” PSU severity, as only experimental or longitudinal designs could test such a research question. Third, our measures involved self-report rather than diagnostic interviewing, and our measures of smartphone use and PSU did not assess objective smartphone use through phone logs (34, 58). Nonetheless, the present study offers important insights into psychological constructs associated with PSU severity and possible mediating variables explaining such relationships.

## Data Availability Statement

The raw data supporting the conclusions of this article will be made available by the authors, without undue reservation.

## Ethics Statement

The studies involving human participants were reviewed and approved by The ethics committee at Tianjin Normal University. The patients/participants provided their written informed consent to participate in this study.

## Author Contributions

HY designed the study protocol. JF conducted data collection and conducted data management, cleaning, and analysis. BL and JF wrote the first draft of the paper. BL and HY substantially revised the manuscript. All authors contributed to the article and approved the submitted version.

## Conflict of Interest

The authors declare that the research was conducted in the absence of any commercial or financial relationships that could be construed as a potential conflict of interest.
